# Persistent cognitive impairment associated with cerebrospinal fluid anti-SARS-CoV-2 antibodies six months after mild COVID-19

**DOI:** 10.1186/s42466-021-00135-y

**Published:** 2021-06-21

**Authors:** Max Borsche, Dirk Reichel, Anja Fellbrich, Anne S. Lixenfeld, Johann Rahmöller, Eva-Juliane Vollstedt, Bandik Föh, Alexander Balck, Christine Klein, Marc Ehlers, Andreas Moser

**Affiliations:** 1 Institute of Neurogenetics, University of Lübeck, Lübeck, Germany; 2 Department of Neurology, University of Lübeck and University Medical Center of Schleswig-Holstein, Campus Lübeck, Ratzeburger Allee 160, 23538 Lübeck, Germany; 3 Outpatient Practice for Neurology and Psychiatry, Lübeck, Germany; 4 Institute of Nutritional Medicine, University of Lübeck and University Medical Center of Schleswig-Holstein, Lübeck, Germany; 5 Department of Anesthesiology and Intensive Care, University of Lübeck and University Medical Center of Schleswig-Holstein, Lübeck, Germany; 6 Department of Medicine I, University Medical Center Schleswig-Holstein, Campus Lübeck, Lübeck, Germany; 7 Center of Brain, Behavior and Metabolism, University of Lübeck, Lübeck, Germany

**Keywords:** COVID-19, SARS-CoV-2, Neurologic sequelae, Cognitive impairment, CSF antibodies

## Abstract

Neurological long-term sequelae are increasingly considered an important challenge in the recent COVID-19 pandemic. However, most evidence for neurological symptoms after SARS-CoV-2 infection and central nervous system invasion of the virus stems from individuals severely affected in the acute phase of the disease. Here, we report long-lasting cognitive impairment along with persistent cerebrospinal fluid anti-SARS-CoV-2 antibodies in a female patient with unremarkable standard examination 6 months after mild COVID-19, supporting the implementation of neuropsychological testing and specific cerebrospinal fluid investigation also in patients with a relatively mild acute disease phase.

**Supplementary Information:**

The online version contains supplementary material available at 10.1186/s42466-021-00135-y.

## Introduction

There is mounting evidence for neurological long-term sequelae after COVID-19 [4]. In the majority of COVID-19 cases exhibiting neurological impairment reported thus far, symptoms were monitored in the acute disease phase and predominantly in hospitalized and severely affected patients [3]. However, neurological long-term impairment after critical illness is a well-established phenomenon in the context of acute respiratory distress syndrome [8], especially when intensive care and ventilation are required. Therefore, it currently remains elusive whether neurological symptoms are rather indirectly related to the infection as a general consequence of critical illness, or whether they are causally related to SARS-CoV-2 infection. Notably, there is evidence for a direct invasion of the central nervous system by the virus [5] and SARS-CoV-2-specific antibodies were detected in the cerebrospinal fluid (CSF) of patients in the acute phase of severe COVID-19 [1]. Potential neurological long-term impairment after mild disease expression has not been systematically studied to date. This latter approach holds the potential to disentangle possible direct virus-mediated sequelae from non-specific persistent disability following critical illness.

## Case description

We report a 57-year-old female with mild but disabling neuropsychological symptoms 6 months after mild COVID-19. During the acute infection, the patient self-referred to a hospital after a positive result of SARS-CoV-2 PCR-testing, which was performed in an outpatient setting due to fever, headache, ubiquitous limb pain, and diarrhea. Upon admittance, vital parameters including peripheral oxygen levels were normal. Laboratory routine examinations were unremarkable despite slightly increased levels of C-reactive protein. Neither intensive care, nor ventilation were required during the hospitalization, and she was discharged home after 5 days. Two weeks after the acute illness, she experienced impaired concentration and action planning. As this condition persisted for several months, preventing her from resuming her professional activity as a nurse and limiting herself in the organization of her activities of daily living, she was referred to our university hospital by a local neurologist. A detailed neurological examination did not detect any abnormalities. In particular, no apraxia or aphasia, and no hypokinetic-rigid signs were found. Apart from a depressive episode in the past, her medical history was unremarkable. A brain MRI examination, standardized testing of the olfactory function (Supplement – BSIT), serologic examination for infectious and autoimmune disease, and further laboratory examinations were likewise normal (Supplement - Laboratory analysis). Workup for both synaptic and paraneoplastic antineuronal antibodies, investigated in serum, was negative (Supplement – Antineuronal antibodies). CSF routine examination was unremarkable, except for a non-specific elevation of CSF protein levels (Supplement – Standard CSF analysis). However, an in-depth standardized neuropsychological assessment revealed relevant impairment regarding “attention”, “alertness” and “working memory”, and the patient scored high on distress and fatigue assessments (Supplementary Table 1). Serologic investigations (Supplement – Methods) revealed positive IgG antibody titers against the S1 (4.2) and the nucleocapsid (NCP) protein (1.7) of SARS-CoV-2 (EUROIMMUN, Lübeck, Germany, values < 0.8 are considered negative, 0.8–1.1 borderline, > 1.1 positive). In comparison to healthy control subjects (*n* = 2), we detected elevated levels of SARS-CoV-2- specific IgG S1 antibodies and a trend towards elevated IgG nucleocapsid antibodies in the CSF (Table 1; Supplement – Methods). Furthermore, as neurotransmitter alterations have been reported in individuals with mild cognitive impairment [6] and animal models of coronavirus infection [2], we investigated levels of gamma-aminobutyric acid (GABA), glutamate, and glutamine in the CSF (Supplement – Methods), but did not observe any significant differences between the index patient and controls subjects (*n* = 3) (Table 1).

**Table 1:** CSF SARS-CoV-2-specific IgG antibodies against the spike (S1) and the nucleocapsid (NCP) protein and neurotransmitter levels in the index patient compared to control subjects

	Index patient	Control subjects	*P* value
CSF SARS-CoV-2 antibodies			
IgG anti-S1, 1:2	0.450	0.044 +/− 0.009 (*n* = 2)	0.017
IgG anti-S1, 1:5	0.190	0.039 +/− 0.009 (*n* = 2)	0.048
IgG anti-NCP, 1:2	0.150	0.036 +/− 0.015 (*n* = 2)	0.102
IgG anti-NCP, 1:5	0.073	0.024 +/− 0.007 (*n* = 2)	0.114
CSF neurotransmitter levels			
Glutamate (μM)	3.70	4.53 +/− 0.82 (*n* = 3)	0.546
GABA (μM)	0.30	0.32 +/− 0.02 (*n* = 3)	0.434
Glutamine (μM)	604	634 +/− 82 (*n* = 3)	0.820

Mean +/− standard deviation is shown. Differences were assessed using student’s t-test. *p* < 0.05 was considered significant

*GABA* Gamma-aminobutyric acid

## Discussion

In conclusion, we demonstrate long-lasting neuropsychologic sequelae along with persistent SARS-CoV-2-specific CSF antibodies several months after a relatively mild acute disease course and encourage the investigation of CSF SARS-CoV-2-specific antibodies and neuropsychological testing to detect possible ‘Long COVID’. Given the mounting evidence for neurological sequelae including cognitive impairment after acute SARS-CoV-2 infection [7], the increasing number of individuals seeking help from neurologists must be expected to become a significant challenge during and after the pandemic. Of note, such complaints were more frequent in, but not limited to severe cases [9], pointing to a relevant role of mild acute SARS-CoV-2 infections in neurological ‘Long COVID’. Our case highlights that investigations beyond routine clinical and laboratory assessment are necessary to identify subtle but clinically relevant sequelae of COVID-19 and that the study of large cohorts for the presence of long-term mild impairment and associated immunological changes is warranted in a longitudinal case-control design.

## Supplementary Information

**Additional file 1.**

## Data Availability

All data generated or analysed during this study are included in this published article and its supplementary information files.

## References

[1] Alexopoulos H, Magira E, Bitzogli K, Kafasi N, Vlachoyiannopoulos P, Tzioufas A, Kotanidou A, Dalakas MC (2020). Anti-SARS-CoV-2 antibodies in the CSF, blood-brain barrier dysfunction, and neurological outcome: Studies in 8 stuporous and comatose patients. Neurology – Neuroimmunology & Neuroinflammation.

[2] Brison E, Jacomy H, Desforges M, Talbot PJ (2011). Glutamate excitotoxicity is involved in the induction of paralysis in mice after infection by a human coronavirus with a single point mutation in its spike protein. Journal of Virology.

[3] Förster, M., Weyers, V., Küry, P., Barnett, M., Hartung, H.-P., & Kremer, D. (2020). Neurological manifestations of severe acute respiratory syndrome coronavirus 2-a controversy ‘gone viral’. *Brain Communications*, *2*(2), fcaa149. 10.1093/braincomms/fcaa149 10.1093/braincomms/fcaa149 PMC7543269 33210085

[4] Josephson SA, Kamel H (2020). Neurology and COVID-19. JAMA.

[5] Matschke J, Lütgehetmann M, Hagel C, Sperhake JP, Schröder AS, Edler C, Mushumba H, Fitzek A, Allweiss L, Dandri M, Dottermusch M, Heinemann A, Pfefferle S, Schwabenland M, Sumner Magruder D, Bonn S, Prinz M, Gerloff C, Püschel K, Krasemann S, Aepfelbacher M, Glatzel M (2020). Neuropathology of patients with COVID-19 in Germany: A post-mortem case series. Lancet Neurology.

[6] Oeltzschner G, Wijtenburg SA, Mikkelsen M, Edden RAE, Barker PB, Joo JH, Leoutsakos JMS, Rowland LM, Workman CI, Smith GS (2019). Neurometabolites and associations with cognitive deficits in mild cognitive impairment: A magnetic resonance spectroscopy study at 7 Tesla. Neurobiology of Aging.

[7] Rass, V., Beer, R., Schiefecker, A. J., Kofler, M., Lindner, A., Mahlknecht, P., et al. (2021). Neurological outcome and quality of life 3 months after COVID-19: A prospective observational cohort study. *European Journal of Neurology*, online ahead of print. 10.1111/ene.14803 10.1111/ene.14803 PMC8250725 33682276

[8] Sasannejad C, Ely EW, Lahiri S (2019). Long-term cognitive impairment after acute respiratory distress syndrome: A review of clinical impact and pathophysiological mechanisms. Critical Care.

[9] Taquet M, Geddes JR, Husain M, Luciano S, Harrison PJ (2021). 6-month neurological and psychiatric outcomes in 236 379 survivors of COVID-19: A retrospective cohort study using electronic health records. Lancet Psychiatry.

