# Supplementary material for: Persistent cognitive impairment associated with cerebrospinal fluid anti-SARS-CoV-2 antibodies six months after mild COVID-19
Source: Neurol Res Pract. 2021 Jun 21;3:34. doi: 10.1186/s42466-021-00135-y (PMC8214919; doi:10.1186/s42466-021-00135-y)
Supplement: Supplementary file 1 — Additional file 1. [file 42466_2021_135_MOESM1_ESM.docx]

**Supplement:**

**Methods:**

***Control samples:*** No history of COVID-19 was known for the control subjects and serum IgG antibody titer against the SARS-CoV-2 spike (S1) and the nucleocapsid (NCP) protein do not indicate any contact with SARS-CoV-2 (EUROIMMUN, Lübeck, Germany).

***Serum and CSF SARS-CoV-2-specific antibody analysis:*** The EUROIMMUN SARS-CoV-2 S1 IgG (#EI 2606-9601-2 G) and the EUROIMMUN SARS-CoV-2 NCP IgG (#EI 2606-9601-2 G) ELISA were performed according to the manufacturer´s instructions. Serum was diluted 1:100 and cerebrospinal fluid (CSF) 1:5 and 1:2.

***Assessment of CSF neurotransmitter levels:*** CSF samples were stored at -80°C until analyses were performed. A total of 80 μL of CSF was diluted with PBS as appropriate and then injected into the high-performance liquid chromatography (HPLC). Every CSF sample was analyzed three times. After pre-column derivatization with o-phthaldialdehyde and sodium sulfite for 30 minutes, we measured glutamate (Glu), glutamine (Gln), and γ-aminobutyric acid (GABA) values using HPLC with electrochemical detection as described previously.^1^ The HPLC system consisted of a C18 column (Eurospher 100, 5 μm, column size 250 . 4 mm) and a pre-column (30 . 4 mm). The isocratic mobile phase (0.1 mol/L PBS, pH 4.5, containing 0.5 mmol/L EDTA and 25% methanol) was previously degassed by helium and pumped at a flow rate of 1.0 mL/min. The compounds were detected electrochemically using a glassy carbon electrode set at a potential of 800 mV relative to an Ag/AgCl reference electrode.

**Further results**

***Laboratory analysis:*** Standard serum analysis in a certified diagnostic laboratory revealed normal total protein levels and the absence of antinuclear antibodies (ANA), anticytoplasmatic antibodies (ANCA), anti-Treponema pallidum and anti-Borrelia antibodies.

***Antineuronal antibodies:*** Serological antibody testing in a certified diagnostic laboratory revealed negative results for antibodies against GAD, CASPR2, GABA-B, LGI 1, NMDA, AMPA, Amphiphysin, CV2, PNMA2, Hu, Ri, Yo, Recoverin, Titin, and DPPX.

***Standard CSF analysis:*** CSF examination including cell count, IgG, IgA, IgM, oligoclonal bands, Amyloid Aß 1-42, Amyloid Aß 1-40, Tau protein, Phospho-Tau protein, and anti-Borrelia antibodies revealed normal results. Only the CSF total protein (59mg/dl, reference range >45mg/dl) and Albumin (36mg/dl, reference range 14-20 mg/dl) were slightly increased.

***BSIT:*** We investigated olfactory function performing the Brief Smell Identification Test (BSIT). Here, the patient scored 10/12 points, leading to an age- and sex-adjusted performance above the 27^th^ percentile, which is considered to indicate normal olfactory function.

| **Global cognitive status** |  |  |
| --- | --- | --- |
| MoCA | 27/30 | cut-off: ≤ 26 |
| **Domain/neuropsychological variable** | **Percentile score**  **(normal range 16-84)** | **Interpretation of results** |
| **Domain: attention and alertness** |  |  |
| TAP alertness without warning ton time/SD | 10/4 | deficit |
| TAP alertness with warning ton time/SD | 2/4 | deficit |
| TAP phasic attention | 2 | deficit |
| TAP divided attention error | 10 | deficit |
| TAP divided attention missing | 10 | deficit |
| TAP go/no go time | 14/5 | deficit |
| TAP go/no go error | 18 |  |
| Trail Making A time | 10 | deficit |
| Trail Making B time | 20 |  |
| **Domain: learning and memory** |  |  |
| Digit span forward WMS-R | 67 |  |
| Digit span backward WMS-R | 88 |  |
| Logical Memory WMS-IV Immediate recall | 25 |  |
| Logical Memory WMS-IV Delayed recall | 16 |  |
| Logical Memory WMS-IV Recognition | 26-50 |  |
| Verbal memory VLMT supraspan (list A) | 70 |  |
| Verbal memory VLMT total list A (learning) | 85 |  |
| Verbal memory VLMT Interference (list B) | 40 |  |
| Verbal memory VLMT consolidation | 85 |  |
| Verbal memory VLMT cued recall | 75 |  |
| Nonverbal memory ROFC immediate recall | 8 | deficit |
| **Domain: executive function - verbal fluency** |  |  |
| Phonemic verbal fluency RWT S-words | 88 |  |
| Phonemic verbal fluency RWT G-R words | 34 |  |
| Semantic verbal fluency RWT animals | 85 |  |
| Semantic verbal fluency RWT cloths-flowers | 58 |  |
| **Domain: executive function - interference** |  |  |
| Reading words FWIT time | 34 |  |
| Naming colors FWIT time | 31 |  |
| Interference FWIT time | 42 |  |
| Planning BADS Key search | Score 5 | intact |
| TAP flexibility total time | 46 |  |
| TAP flexibility total error | 69 |  |
| TAP flexibility Index speed accuracy | 50 |  |
| TAP flexibility Index total | 69 |  |
| TAP working memory time | 8 | deficit |
| TAP working memory missing | 27 |  |
| **Domain: visuospatial abilities** |  |  |
| copy 3-d figures |  | intact |
| ROFC copy | > 50 |  |
| **Emotional state** |  |  |
| BSCL Global GSI | 94 | high distress |
| BSCL Global PST | 88 |  |
| BSCL Global PSDI | 97 | number of items high |
| **Fatigue Scale** |  |  |
| WEIMuS general fatigue score | 99.6 | Severe |
| WEIMuS mental fatigue/physical fatigue score | 99/99 | Severe |

***Supplementary Table 1: Neuropsychological test results***

A right-handed 57-year-old female subject with 17 years education was examined by a substantial neuropsychological test battery. Domains, specific tests, test results, and interpretation of findings are shown. MoCA: Montreal Cognitive Assessment^2^; TAP: test battery for testing of alertness (Testbatterie zur Aufmerksamkeitsprüfung^3^); WMS-R: Wechsler Memory Scale (revised version)^4^; VLMT: Verbaler Lern- und Merkfähigkeitstest^5^; RWT: Regensburger Wortflüssigkeitstest^6^; FWIT: German version of the Stroop test: Farb-Wort-Interferenztest^7^; BADS: Behavioral Assessment of the Dysexecutive Syndrome^8^; ROFC: Rey-Osterrieth Complex Figure^9^; WEIMuS – Würzburger Erschöpfungsinventar bei MS^10^; BSCL: Brief-Symptom-Checklist^11^

***Supplementary References***

1. Prauss K, Varatharajan R, Joseph K, Moser A. Transmitter self-regulation by extracellular glutamate in fresh human cortical slices. J Neural Transm 2014;121:1321–1327.

2. Nasreddine ZS, Phillips NA, Bédirian V, et al. The Montreal Cognitive Assessment, MoCA: a brief screening tool for mild cognitive impairment". J Am Geriatr Soc 2005;53 (4): 695–9.

3. Zimmermann P, Fimm B. Testbatterie zur Aufmerksamkeitsprüfung (TAP). Version 2.2. PsyTest; 2009.

4. Härting C, Markowitsch, H-J, Neufeld N, et al. Wechsler Memory Scale – Revised Edition, German Edition. Huber, 2000.

5. Helmstaedter C, Lendt M, Lux S. Verbaler Lern- und Merkfähigkeitstest (VLMT). Göttingen: Hogrefe, 2001.

6. Aschenbrenner S, Tucha O, Lange KW. Regensburger Wortflüssigkeitstest (RWT). Göttingen: Hogrefe, 2000.

7. Bäumler G. Farbe-Wort-Interferenztest (FWIT). Göttingen: Hogrefe, 1985.

8. Wilson BA, Emslie H, Evans JJ, et al. Behavioural Assessment of the Dysexecutive Syndrome (BADS). Bury St Edmunds: 2000.

9. Osterrieth PA. Le test de copie d’une figure complexe. Arch Psychol 1944: 30:206–356.

10. Flachenecker P, König H, Müller G, et al. Würzburger Erschöpfungs-Inventar bei Multipler Sklerose (WEIMuS). Würzburg: 2003.

11. Franke, G.H., Brief-Symptom-Checklist (BSCL). Göttingen: Hogrefe, 2017
